# Impact of Preoperative Serum Albumin Level on the Outcome of Colorectal Cancer Surgery

**DOI:** 10.7759/cureus.57655

**Published:** 2024-04-05

**Authors:** Abdulaziz Alajmi, Abdullah Almehari, Ali R Alzahrani, Yazeed Aljurays, Nawaf Alzahrani, Abdulellah M Aladel, Nayef Alzahrani

**Affiliations:** 1 Medicine, King Saud Bin Abdulaziz University for Health Sciences College of Medicine, Riyadh, SAU; 2 Mathematics Department, Faculty of Sciences, Umm Al-Qura University, Makkah, SAU; 3 General Surgery, King Abdulaziz Medical City, Riyadh, SAU; 4 General Surgery, King Saud Bin Abdulaziz University for Health Sciences, Riyadh, SAU

**Keywords:** postoperative outcome, hypoalbuminemia, complications, colorectal cancer surgery, albumin

## Abstract

Background

Gastrointestinal malignancy surgeries are known to have a risk of postoperative complications. Preoperative nutritional status has been suggested as a potential predictor of postoperative outcomes, with low serum albumin levels utilized as a marker of malnutrition and increased risk of postoperative complications. This paper investigated the association between preoperative serum albumin levels and postoperative outcomes in patients undergoing colorectal cancer surgery.

Methods

This retrospective data-maintained study was based on all patients aged 18 years and above who underwent colorectal cancer surgery at King Abdulaziz Medical City, Riyadh, Saudi Arabia between 2015 and 2022.

Results

A total of 400 patients were included in the study. With an average age of 64.43 years. Males represented 254 (63%) of the patients, while females accounted for 146 (37%). Thirty percent of patients had hypoalbuminemia (i.e., albumin level below 35 g/L) before surgery. Among the sample, 112 (28%) experienced complications after surgery. The mean albumin level for patients who experienced postoperative complications was 30.46 g/L while patients without complications had a normal albumin level. As for the length of hospital stay, it was eight days for patients with a normal albumin level and 23 days for hypoalbuminemia patients.

Conclusion

In conclusion, preoperative hypoalbuminemia is associated with poor patient outcomes and can be utilized as a prognostic marker for patients in need of colorectal cancer surgery.

## Introduction

Colorectal cancer (CRC) is a common malignancy in Saudi Arabia [1,2]. CRC has an age-standardized rate (ASR) of 13.9 per 100,000 in males and an ASR of 11.3 per 100,000 in females in the population of Saudi Arabia [3]. The mainstay of treatment choice for CRC is surgery [4]. However, major abdominal surgeries are accompanied by a significant likelihood of adverse events, such as infections, bleeding, and organ dysfunction after surgery [5,6]. Preoperative nutritional status has been suggested in the literature as a potential predictor of post-surgical outcomes, with low serum albumin levels being proposed as an indicator of poor nutritional status and an increased risk of poor outcomes after the operation [7]. Serum albumin is the most prevalent circulating protein, which can make up as much as half of the total protein content found in the plasma [8]. Albumin is synthesized and secreted by hepatocytes into the body's circulation to aid in the regulation of the body's oncotic pressure and transportation of ligands such as steroids, fatty acids, and thyroid hormones [8]. However, this relationship between serum albumin levels and postoperative outcomes has been questioned in the literature. For instance, a study published in 2017 suggests that no direct relationship exists between low albumin levels alone and poor outcomes after the operation [9]. The study even argues that interventions such as intravenous albumin infusion, aimed at correcting hypoalbuminemia, did not significantly alter the course of the patient's hospital stay [9]. Therefore, our study aims to investigate the association between preoperative serum albumin levels and postoperative outcomes in patients undergoing CRC surgery.

## Materials and methods

This study was conducted on all patients who underwent CRC surgery at King Abdulaziz Medical City, Riyadh, Saudi Arabia after getting approval from the Institutional Ethics Committee named King Abdullah International Medical Research Center (KAIMRC) with a study number (NRC23R/679/10). The aim of our study was to investigate the association between preoperative serum albumin levels and postoperative outcomes in patients undergoing CRC surgery.

Primary objectives

The primary objective of the study was to determine whether low preoperative serum albumin levels were associated with an increased risk of postoperative complications such as wound infection, urinary tract infection, pneumonia, anastomosis leak, and sepsis. Another outcome of interest was the length of hospital stay, time spent in ICU, and 90-day mortality rate in patients undergoing CRC surgery.

Secondary objectives

Secondary outcomes were to determine the prevalence of low preoperative serum albumin levels (serum albumin < 35.00 g/L) in patients undergoing CRC surgery.

Study design

A retrospective cohort study was conducted for a duration of four months, involving a sample size of 400 patients who met the inclusion and exclusion criteria. Data was collected from medical electronic records using the BESTCare system. After that, the data was cleaned and revised for any potential errors.

Inclusion and exclusion criteria

The study population comprised patients aged 18 years and above, admitted for either emergency or elective reasons. The study included all patients enrolled between March 2015 and August 2022. However, patients diagnosed with liver disease prior to the study, or those who received nutritional support or albumin supplementation before surgery, were excluded from the study. Additionally, all patients with missing data were also excluded from the study.

Data analysis

The extracted data was analyzed using SPSS version 28. The study reported the mean, standard deviation, median, and range for continuous variables such as age, height, length of hospital stays, days spent in the ICU, weight, and albumin level. Additionally, the frequency and percentage were reported for categorical variables such as sex, comorbidities, smoking habits, postoperative complications, mortality, and BMI. The association between preoperative serum albumin levels and postoperative outcomes was examined using ANOVA and the T-test for categorical variables. Linear regression was used for continuous variables to investigate the association between preoperative serum albumin levels and postoperative outcomes, controlling for potential confounding factors such as length of hospital stay, sex, and mortality that may affect the association between preoperative serum albumin levels and postoperative outcomes.

## Results

The study encompassed a total number of 400 participants, with a gender distribution of 146 (37%) females and 254 (63%) males. The mean preoperative serum albumin level for males was 36.62 g/L, with 67 (54.9%) of male patients classified as hypoalbuminemic (i.e., less than 35.00 g/L). Conversely, the mean preoperative serum albumin level for females was 35.4 g/L, with 55 (45.1%) of female patients classified as hypoalbuminemic (Table 1).

**Table 1:** Background characteristics of low- and normal albumin groups. SD*: standard deviation

	Albumin <35 n (%)	Albumin ≥35 n (%)
Total	122 (30.5)	278 (69.5)
Male	67 (54.9)	187 (67.3)
Female	55 (45.1)	91 (32.7)
Smoker	18 (14.8)	36 (12.9)
Comorbidities	97 (79.5)	208 (74.8)
Mortality	6 (4.9)	0
Had Postoperative complications	102 (83.6)	10 (3.6)
Age (years) mean ± SD^*^	69.55±12.9	62.18±11.81
BMI mean ± SD^*^	27.95±6.88	28.37±5.62
ICU (days) mean ± SD	3.19±6.12	0.09±1.106
Length of stay at hospital (days) mean ± SD	22.65±16.79	8.26±3.01

A T-test was conducted to ascertain any statistical difference between genders regarding average serum albumin levels. The results indicated a statistically significant difference between males and females, with a p-value of 0.013 (< 0.05), leading to the conclusion that male albumin levels are higher than those of females (Appendices Table 4). Moreover, an ANOVA test against albumin level demonstrated a significant difference in the mean albumin level of the no-complications group among most of the 22 groups of postoperative complications (Appendices Table 5). A subsequent T-test, which grouped all complications as one value and no complications as another, confirmed the ANOVA test result (Appendices Table 6). The mean average albumin level for patients who experienced postoperative complications was 30.46 g/L, indicative of hypoalbuminemia, while patients without complications had a normal albumin level (Table 2). 

**Table 2:** Tukey's Honest Significant Difference (Post-Hoc) comparisons between no postoperative complications against 22 groups of postoperative complications. *the mean albumin level for these combined complications is statistically significant (check the p-value of it).

(I) Postoperative Complications	(J) Postoperative Complications	Mean Difference (I-J)	Std. Error	P-value	95% CI	
					Lower Bound	Upper Bound
No complications	Wound infection	4.983^*^	1.211	0.009^*^	.57	9.40
	Urinary tract infection	4.861^*^	1.166	0.008^*^	.61	9.11
	Wound infection + urinary tract infection	7.837^*^	1.056	<0.001^*^	3.99	11.68
	Pneumonia	8.199^*^	1.855	0.003^*^	1.44	14.95
	Wound infection + pneumonia	9.399^*^	1.211	<0.001^*^	4.99	13.81
	Urinary tract infection + pneumonia	13.799^*^	1.855	<0.001^*^	7.04	20.55
	Wound infection + urinary tract infection + pneumonia	9.399^*^	2.070	0.002^*^	1.86	16.94
	Anastomosis leak	5.971^*^	1.573	0.030^*^	.24	11.70
	Wound infection + anastomosis leak	8.399	2.386	0.073	-.29	17.09
	Urinary tract infection + anastomosis leak	4.399	2.917	0.998	-6.23	15.03
	Wound infection + urinary tract infection + anastomosis leak	8.899	2.917	0.254	-1.73	19.53
	Wound infection + pneumonia + anastomosis leak	9.399	2.917	0.169	-1.23	20.03
	Sepsis	7.066	2.386	0.309	-1.63	15.76
	Wound infection + sepsis	8.899	2.917	0.254	-1.73	19.53
	Urinary tract infection + sepsis	9.149^*^	2.070	0.003^*^	1.61	16.69
	Pneumonia + sepsis	7.733	2.386	0.161	-.96	16.42
	Wound infection + pneumonia + sepsis	14.399^*^	2.917	<0.001^*^	3.77	25.03
	Urinary tract infection + pneumonia + sepsis	11.066^*^	2.386	0.001^*^	2.37	19.76
	Anastomosis leak + sepsis	3.899	2.917	1.000	-6.73	14.53
	Wound Infection + anastomosis leak + sepsis	10.233^*^	1.696	<0.001^*^	4.06	16.41
	Wound infection + urinary tract infection + anastomosis leak + sepsis	7.399	2.917	0.626	-3.23	18.03
	Wound infection + urinary tract infection + pneumonia + anastomosis leak + sepsis	11.899^*^	2.917	0.011^*^	1.27	22.53

A T-test between albumin level classification and the length of hospital stay showed a significant difference between the means of length of hospital stay between patients with a normal albumin level and those with hypoalbuminemia. The length of hospital stay was eight days for patients with a normal albumin level and 23 days for hypoalbuminemia patients. As for time spent in the ICU, it was one day for patients with normal albumin levels and three days for patients with low albumin levels (Appendices Tables 6-7). Finally, a linear regression analysis was conducted to predict postoperative complications on several factors: the duration of hospital stays, mortality rate, albumin level, and gender. Each of these variables significantly influenced the prediction of postoperative complications except gender. An increase in the values of albumin level was associated with a decrease in postoperative complications. Oppositely an extended length of hospital stays correlated with an increase in postoperative complications. Gender was not a significant factor. This pattern was also observed in relation to mortality. Furthermore, the serum albumin levels for patients who survived were four times higher than those who did not survive. However, the length of stay in the ICU and the smoking status of patients were not statistically significant, likely due to their low representation in the data set. Moreover, higher postoperative complications are associated with higher mortality (Table 3).

**Table 3:** Linear regression model. Postoperative complications are dependent variables and independent variables are gender, length of stay, mortality, preoperative albumin level. ^a^Dependent variables

Coefficients^a^						
Model		Unstandardized Coefficients		Standardized Coefficients	t	p-value
		B	Std. Error	Beta		
1	(Constant)	20.662	2.619		7.889	<0.001
	Gender	-0.620	0.507	-0.050	-1.224	0.222
	Length of hospital stay (days)	0.163	0.024	0.318	6.915	<0.001
	Preoperative serum albumin level	-0.351	0.051	-0.323	-6.874	<0.001
	Mortality (90 days) yes/no	-7.236	2.039	-0.147	-3.549	<0.001

## Discussion

Surgical intervention is a major treatment option available for patients with CRC that can present a variety of unpredictable outcomes for every individual patient. Thus, accurately predicting the postoperative prognosis and complications is crucial for a more personalized care plan, allowing for an optimal stratification of patients who might require intensive monitoring afterward. Our paper investigated the connection between low serum albumin levels prior to surgery and postoperative outcomes in patients who underwent CRC surgery. Our study found that hypoalbuminemia was significantly linked with a higher risk of negative outcomes after surgery such as surgical site infection, urinary tract infection, and increased length of hospital stays. Based on our study’s findings, we can conclude that preoperative serum albumin level is a useful prognostic biomarker for CRC patient outcomes after surgery. These findings are aligned with previous studies in the literature that have shown the association between hypoalbuminemia and poor surgical outcomes [10-12]. One example of such studies is a paper that analyzed patients who have undergone significant gastrointestinal surgery [13]. It included a total of 670 patients, for which around half of them had a normal albumin level (i.e., equal to or greater than 35 g/L), and the other half had hypoalbuminemia (i.e., less than 35 g/L) [13]. The results demonstrated that patients with low preoperative serum albumin levels had a higher postoperative mortality and duration of stay after operation [13]. As for individuals with severe hypoalbuminemia (i.e., less than 20 g/L), their risk of postoperative mortality was around seven to eight times greater and required stays in the hospital of six to eight days longer [13]. In addition, a paper published in 2016 has concluded that a correlation does exist between low levels of albumin prior to surgery and suboptimal outcomes in the perioperative period [14]. An explanation for this association could be that albumin is linked to the nutritional status of patients [15]. Consequently, low preoperative albumin levels indicate a state of malnutrition, advanced disease status, and increased risk for postoperative complications [16]. Malnourishment in particular is described in the literature as a prognostic factor for unfavorable outcomes in CRC patients [17]. Moreover, the nature of GI dysfunction in CRC particularly in the loss of normal absorption and metabolism secondary to cancer infiltration to the gastrointestinal cell walls puts CRC patients at a stronger likelihood for low state of nutrition [18]. Therefore, the preoperative measurement of serum albumin levels may reflect the severity of the patient’s condition and predict the surgical outcomes. Furthermore, we observed an interesting finding regarding the relationship between serum albumin levels and gender. Our analysis indicated that hypoalbuminemia was more prevalent in females. Interestingly, the existing literature presents conflicting data on this issue, with some studies associating hypoalbuminemia with the male gender [11]. On the other hand, other studies have noted this association but with the female gender [19]. Although the cause of this association remains unclear, one study that analyzed the serum albumin concentration in over a million samples found that the mean level of albumin decreases more rapidly in females than in males and attributed these findings to a potential hormonal influence [20]. It is also important to note, however, that our study focused on CRC surgery outcomes and did not explore this association with other types of surgeries or malignancies. Previous studies have also primarily linked the association between hypoalbuminemia and poor outcomes to CRC surgery [21-23]. Therefore, the proposed association between low preoperative serum albumin levels and poor patient outcomes may be specific only to the surgical procedure and patient population studied and not conversely applicable to other types of cancer like lung, kidney, or breast carcinoma. To the best of our knowledge, this paper is the first study describing the effect of hypoalbuminemia on postoperative CRC outcomes in Saudi Arabia and the Middle East as a whole. Thus, the implications of our findings are multiple. Firstly, it suggests that preoperative serum albumin levels may be a reliable indicator for predicting patient outcomes after surgery in patients with CRC. The study's findings demonstrate the importance of identifying patients at an increased risk of postoperative complications who may benefit from preoperative nutritional counseling. The study's results also seek to help in the development of standardized guidelines for preoperative nutritional assessment and management for patients undergoing CRC surgery. Secondly, our study highlights the need for further research to better understand the relationship between serum albumin levels and gender and other prognostic factors that might influence patient outcomes after CRC surgery. Future studies could also investigate other potential markers or predictors of surgical prognosis, as well as explore the impact of different surgical techniques on patient outcomes. Nevertheless, health providers should utilize other factors as well in the context of determining the prognosis of CRC patients, such as active smoking history, tumor stage, presence of metastasis on imaging, and the patient’s overall health status when making management decisions.

Study limitations

There are several limitations. In our study, we excluded all CRC patients with incomplete data in the electronic health records from the data analysis. This exclusion criterion inevitably reduced the sample size utilized in our analysis. Furthermore, the study was conducted in a single center, which inherently limited the sample size included in our research. Consequently, these factors may have introduced a potential bias in our sample, thereby reducing the generalizability of our findings. It is important to consider these limitations when interpreting the results of our study.

## Conclusions

Overall, our study contributes to the existing knowledge that preoperative hypoalbuminemia is associated with unfavorable patient outcomes after CRC operations. It signifies the utilization of albumin levels as a biomarker that can help in identifying patients with a high postoperative risk. The study also emphasizes the importance of considering multiple patient factors and individual patient characteristics when making surgical decisions and evaluating postoperative outcomes in CRC patients. We recommend future studies to also assess the long-term survival outcomes and investigate other potential contributing factors that could play a role in CRC surgery outcomes.

## Appendices

As mentioned in the Results section, this table shows the statistical difference between genders regarding average serum albumin levels (Table 4).

**Table 4:** The difference in albumin average levels between males and females.

		Levene's Test for Equality of Variances			95% Confidence interval	
		F	Sig.	t	Lower	Upper
Preoperative serum albumin level	Equal variances assumed	6.291	0.013	-2.14	-2.34	-.0102
	Equal variances not assumed			-2.03	-2.41	-0.038

In addition, the next table shows the difference in the mean albumin level of the no-complications group against the 22 groups of postoperative complications (Table 5).

**Table 5:** ANOVA test shows the difference in the mean albumin level of the no-complications group against the 22 groups of postoperative complications.

	Sum of Squares	df	Mean Square	F	P-value
Between groups	5801.288	22	263.695	15.600	<0.001
Within groups	6372.462	377	16.903		
Total	12173.750	399			

Moreover, the next table shows the albumin level classification and length of the stay at the hospital (Table 6).

**Table 6:** T-test between albumin level classification and length of the stay at hospital.

Group Statistics					
	Preoperative serum albumin level (albumin level) >=35 or <35 (g/L)	N	Mean	Std. Deviation	Std. Error Mean
Length of hospital stay (days)	Hypoalbuminemia	122	22.65	16.795	1.521
	Normal albumin	278	8.26	3.006	0.180

To add on to the previous table is this table showing the independent sample tests of both the hospital stay and time spent in the ICU (Table 7).

**Table 7:** The length of stay between the hypoalbuminemia group and normal albumin level.

Independent Samples Test											
		Levene's Test for Equality of Variances		T-test for Equality of Means							
		F	Sig.	t	df	Significance		Mean Difference	Std. Error Difference	95% Confidence Interval of the Difference	
						One-Sided p	Two-Sided p			Lower	Upper
Length of hospital stay (days)	Equal variances assumed	66.97	<0.001	13.81	398	<0.001	<0.001	14.38	1.042	12.340	16.437
	Equal variances not assumed			9.39	124.41	<0.001	<0.001	14.38	1.531	11.358	17.419
Time spent in ICU (days)	Equal variances assumed	118.75	<0.001	8.04	398	<0.001	<0.001	3.09	0.385	2.341	3.856
	Equal variances not assumed			5.47	124.37	<0.001	<0.001	3.09	0.567	1.977	4.220
